# Role of GPR39 in Neurovascular Homeostasis and Disease

**DOI:** 10.3390/ijms22158200

**Published:** 2021-07-30

**Authors:** Yifan Xu, Anthony P. Barnes, Nabil J. Alkayed

**Affiliations:** 1 Department of Anesthesiology and Perioperative Medicine, Oregon Health and Science University, Portland, OR 97239, USA; xyi@ohsu.edu; 2 Knight Cardiovascular Institute, Oregon Health and Science University, Portland, OR 97239, USA; barnesan@ohsu.edu

**Keywords:** GPR39, zinc, eicosanoids, biased agonist, dementia, vascular tone, depression, epilepsy

## Abstract

GPR39, a member of the ghrelin family of G protein-coupled receptors, is zinc-responsive and contributes to the regulation of diverse neurovascular and neurologic functions. Accumulating evidence suggests a role as a homeostatic regulator of neuronal excitability, vascular tone, and the immune response. We review GPR39 structure, function, and signaling, including constitutive activity and biased signaling, and summarize its expression pattern in the central nervous system. We further discuss its recognized role in neurovascular, neurological, and neuropsychiatric disorders.

## 1. Introduction

G-protein coupled receptors (GPCRs) are one of the largest gene families in the genome with diverse functions throughout the body. GPCRs share a canonical seven transmembrane (7TM) architecture that allows them to detect a variety of cellular stimuli, including those associated with sensory stimuli (e.g., vision and taste) as well as those produced within the body (e.g., humoral factors, cytokines, and neurotransmitters), often acting to shape and balance physiologic responses at the cellular or tissue level. Their ability to sense small molecules also makes this class of receptor ideal targets for pharmacological interventions, with more than a third of current FDA-approved drugs targeting GPCRs [1,2]. Many GPCRs have identified endogenous ligands and, in some cases, well-characterized synthetic ligands that are employed as therapeutics. A subset of GPCRs remain enigmatic without clear cognate ligands and are referred to as ‘orphan’ receptors [1]. Despite our limited knowledge of how such receptors are engaged, a combination of genetic loss-of-function studies and receptor-selective compounds derived from high-throughput screening have established critical roles for many of these orphan receptors in normo- and patho-physiology.

One such receptor is GPR39, which has been linked to a number of potential ligands, including Obestatin, a ghrelin-related peptide proposed to be a natural ligand for GPR39, but the physiologic role of Obestatin remains controversial [3,4]. Another candidate ligand is the essential micronutrient zinc, which serves as a cofactor and messenger for a number of critical cellular functions. Recent studies have suggested that zinc at typical physiologic concentrations tends to act as a modulator, rather than an activator, of GPR39 [5,6]. Our efforts to understand lipid signaling in the cardio- and cerebro-vascular systems has recently revealed that GPR39 is a dual sensor for two eicosanoids with opposing actions on microvascular tone: the vasodilator 14,15-epoxyeicosatrienoate (14,15-EET) and vasoconstrictor 15-hydroxyeicosatetraenoate (15-HETE) [5]. We have also implicated GPR39 in aging-related vascular cognitive impairment and dementia (VCID; [7]). This review summarizes the basic biology of GPR39, its expression and regulation of the mammalian nervous system, and highlights its homeostatic role in maintaining vascular tone, inflammatory balance, and modulating neural circuit excitability. It is through these mechanisms that GPR39 can impact neurologic, neuropsychiatric, and neurovascular pathologies, emphasizing its potential as a therapeutic target.

## 2. Gene Organization and Isoforms of GPR39

**Figure 1:**
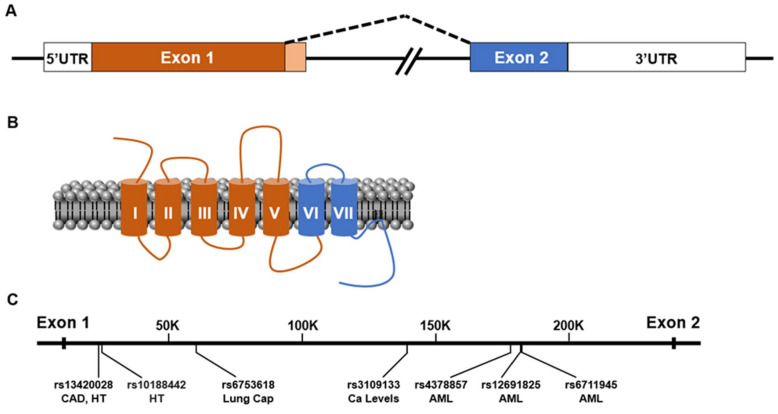
GPR39 gene organization and single nucleotide polymorphisms. (**A**). Diagram indicating the production of GPR39 transcripts. GPR39-1a is produced by splicing of Exon 1 (orange) and Exon 2 (blue), and GPR39-1b is produced by transcriptional intronic read-through of Exon 1 (light orange) that contributes an alternative carboxy terminus sequence. (**B**). Illustration of GPR39 protein as a seven transmembrane protein with color coding of exon contribution to the protein (orange, Exon 1 and blue, Exon 2). (**C**). Diagram of single nucleotide polymorphisms of GPR39 associated with phenotypes, coronary artery disease (CAD), hypertension (HT), lung capacity (Lung Cap), calcium levels (Ca levels), and acute myeloid leukemia (AML).

Low stringency hybridization cloning using Ghrelin receptor (GhrelinR/GHS-R) cDNA identified two orphan GPCRs, GPR38 and GPR39, in a human brain cDNA library [8]. This route of discovery is consistent with protein homology comparisons that place GPR39 in the Ghrelin receptor family of peptide-activated GPCRs that include receptors for the neuropeptides Ghrelin, Neurotensin, Neuromedin U, and Motilin. GPR39 is widely expressed in multiple organ systems, including the central and peripheral nervous systems [8,9,10,11]. The human *gpr39* gene spans 230 kilobases located on chromosome 2q21-q22 [8], while mouse *gpr39* resides on chromosome 1. In the genomes examined, the GPR39 gene’s last exon overlaps with the antisense last exon of the Lypd1 gene on the antisense strand [12]. The positional relationship of these two genes may explain the somewhat complementary expression patterns observed for each [12]. The GPR39 locus encodes two exons that produce two splice variants (Figure 1A,B). One variant (GPR39-1a) includes both exons and produces the full-length receptor with the typical 7-transmembrane (7-TM) architecture associated with GPCRs. In published literature, this isoform responds to a number of natural and synthetic ligands through the activation of downstream signaling pathways, such as those found in neuronal synaptic transmission [13,14,15]. The second isoform (GPR39-1b) is produced by a transcript that includes only the first exon of the locus and encodes a protein that contains only the first five TM domains of GPR39, lacking the remaining two TM domains, last extracellular (ECL) loop, and carboxy tail of the receptor. Consistent with this limited structure, the GPR39-1b isoform has been reported to lack zinc ligand activation [16]. These truncated splice variants are observed for all members of the ghrelin receptor family, including the truncated receptors for Neurotensin-1 (NTSR-1) [17] and Ghrelin (GnR) observed to be expressed in the CNS [18]. The truncated GnR has been implicated to buffer full length receptor function in a concentration-specific manner: highly expressed truncated GnR decreases full length GnR signaling [18,19], while low concentrations of truncated GnR increases full length GnR trafficking to the plasma membrane [20] through dimerization. Interestingly, GPR39-1b does not dimerize with GPR39-1a, but it can dimerize with NTSR1 and decrease its signaling [21].

## 3. GPR39 Expression Patterns

Several studies have explored GPR39 expression; they are discussed below and summarized in Table 1A. Northern blots of human tissue RNA indicate that GPR39-1a mRNA is expressed in the gastrointestinal tract, spleen, lung, heart, and reproductive and adipose tissue, while GPR39-1b exhibits a broader expression pattern that includes stomach, small intestine, colonocyte epithelium, and multiple brain regions (frontal cortex, septum, amygdala, and hippocampus, but not the hypothalamus) [8,9,11]. In situ hybridization of mouse brain found the highest GPR39 mRNA expression in the amygdala, hippocampus (dentate gyrus, CA1, CA3), and auditory cortex [10]. This study also noted lower expression in piriform cortex, ventral pallidum, and inferior olive and confirmed a lack of hypothalamic expression [10]. However, in the rat brain, GPR39 mRNA has been detected at very low levels in the hypothalamus using real-time RT-PCR [11]. GPR39 mRNA expression in lateral amygdala (fear perception, conditioning) and ventral hippocampus CA1 (memory and learning) of both rodents and humans supports a role for GPR39 in seizures, as well as neuropsychiatric disorders involving stress, sensory processing, memory, and emotional processing. At the cellular level, GPR39 has been described in postsynaptic membranes, where it plays a role in regulating presynaptic glutamate release [22,23].

It is important to note that significant differences in GPR39 expression patterns have been reporter-specific between both species and detection methods. As mentioned above, real-time RT-PCR in rats showed that GPR39-1b is expressed widely in the CNS in one study, whereas GPR39-1a is expressed only at a level slightly above detection [11,12]. However, in situ hybridization detects strong expression of GPR39-1a in mouse hippocampus and amygdala [10]. On a cell-specific level, using immunohistochemistry against the third extracellular domain of GPR39-1a in human dorsolateral prefrontal cortex, Davis and colleagues [7] detected protein expression in microglia and peri-capillary cells resembling pericytes, with GPR39-positive microglia increasing in density in brains from deceased individuals with a history of cognitive-impairment. Proteomics studies have shown protein expression to be highly expressed in the circulatory system as well as the blood, heart, lung, immune system, and endocrine systems. Microarray transcriptome and RNAseq studies, on the other hand, have demonstrated wide transcriptional expression in the brain (cerebral cortex and prefrontal cortex), the spinal cord, the heart, the gastrointestinal system, and the immune system, as well as muscle, skin, lung, and reproductive organs (https://www.proteomicsdb.org/proteomicsdb/#protein/proteinDetails/48809/expression available on 30 July 2021). GPR39’s relatively low expression levels in some brain regions complicates the use of single cell RNA sequencing (sc-RNAseq) studies to assign cell-type specific patterns, but high expression of GPR39 mRNA in glutamatergic neurons have been reported, while GABAergic and glial cells showed little or no GPR39 [15]. As the sensitivity of sc-RNAseq improves, a clearer picture of GPR39 expression will undoubtedly be resolved.

## 4. Preliminary GWAS Data Suggests Potential Roles for GPR39 in Human Vascular and Neurovascular Disease

Multiple single nucleotide polymorphisms (SNPs) have been identified in GPR39 through large scale GWAS studies that link the receptor with intronic risk alleles that are associated with diverse pathologies. Linked diseases currently include: coronary artery disease (CAD; rs13420028), hypertension (rs13420028; rs10188442), acute myeloid leukemia development (rs6711945; rs4378857; rs12691825), lung capacity in smokers (rs6753618), and altered calcium levels in the east Asian population (rs3109133) (based on the GWAS Catalog (National Human Genome Research Institute-European Bioinformatics Institute)) (Figure 1C). No SNPs have been identified in GPR39 specifically for nervous system disorders. However, neurons are the most metabolically sensitive cells in the body and require a delicate supply of nutrients and oxygen from healthy blood vessels for survival. Among the conditions above, CAD, hypertension, smoking, and calcium levels are interrelated through vascular disease and, thus, may lead to dramatic consequences for brain health. A recent study applied sequence kernel association testing to rare GPR39 polymorphisms [28] and was able to link combination of loss-of-function, missense, and changes in codon usage to International Classification of Diseases (ICD) codes associated with the DiscovEHR cohort [29], with the most significant linkage to ICD354.2 (lesion of the ulnar nerve). Additional codes were identified parallel to some of the GWAS findings and included a variety of conditions: benign prostate hyperplasia, benign essential hypertension, cardiovascular disease, and macular puckering of retina. This work was also able to evaluate a subset of these alterations in the context of GPR39 signaling and receptor surface expression, indicating that individual amino acid substitutions in the receptor have profound effects on its function. Interestingly, GPR39 mouse knockout studies (Table 2) have also implicated the receptor in wound healing, as well as bone and gastrointestinal homeostasis [30,31,32,33,34]. Taken together, these studies from human genetics point to the potential of GPR39 to contribute to, or modify, disease processes.

## 5. G-protein Regulation via Post-Translational Modification

GPR39-1a appears to possess both constitutive activity and ligand-induced activation, with the latter inversely related to constitutive activity. Evidence also exists for ligand-specific biased agonism for differential downstream G-protein signal transduction [41,42]. Post-translational modifications appear to increase ligand-induced G-protein activity at the expense of constitutive G-protein activity. For instance, a prototypical disulfide bond bridges Cys108 in transmembrane domain III (TMIII) and Cys 210 in the second extracellular domain (ECL-2). Mutagenesis disrupting this bond decreases GPR39 cell surface expression and eliminates zinc-induced, Gαq-mediated inositol phosphate production but also increases constitutive signaling through Gα12/13 [43]. A second, non-canonical disulfide bond exists between Cys11 in the N terminus domain and Cys191 in ECL-2, and the loss of this bond increases zinc potency ten-fold. This finding has led to the proposal that this second disulfide bridge covalently ‘gates’ across the entrance to the main ligand binding pocket, analogous with the structure found in the beta-2 adrenergic-like receptors, which attenuate ligand access and receptor activity [43].

GPR39 also contains other established post-translational modifications that are established activity modifiers in other GPCRs. ^32^P labeling studies indicate that GPR39 is phosphorylated [44], and this likely includes phosphorylation at both serine and threonine residues. Some of these sites have been confirmed via mass spectrometry and are predicted to be modified by multiple kinases,. The receptor also contains predicted sites for palmitoylation (360 and 361, human), and N-linked glycosylation is predicted to occur on asparagine residues 192, 206, and 212 (human). While the impact of these sites has not been tested in the case of GPR39, such modifications often contribute to GPCR trafficking, turnover, and signaling. Indeed, a study replacing the less phosphorylated carboxy tail of GnR with the highly phosphorylated carboxy tail of GPR39 results in elimination of GnR’s basal internalization and decreases GnR’s agonist-induced internalization in HEK293 cells [44].

## 6. Constitutive Pathway Acts through Gα12/13 and Rho Kinase to Promote Resistance to Stress and Inflammation

**Figure 2:**
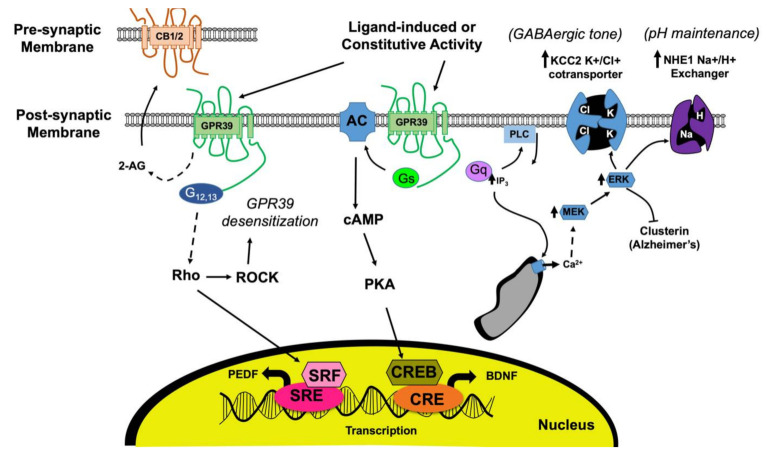
GPR39 signaling cascade in neural pathology. GPR39 activation by ligands activate the Gαq and Gαs pathways. Gαq activation results in PIP2 cleaving to release membrane-bound DAG and cytosolic IP3, resulting in calcium release from ER to increase KCC2 and promote GABAergic tone, increase NHE1 activity to maintain pH, and increase 2-AG synthesis to increase presynaptic CB1R activity. ERK1/2 activation from this signaling pathway has been shown to inhibit clusterin, a protein associated with Alzheimer’s disease. Gαs signaling increases cAMP levels and CRE-dependent transcription, including BDNF and TrkB, which are associated with improved memory and decreased depression. This pathway is inhibited through PKA by PKIB from the constitutive pathway of GPR39, which is coordinated through Gα12/13 and Rho kinases for SRE-dependent transcription, promoting anti-oxidative and anti-inflammatory activity. The constitutive pathway also has negative feedback for GPR39 expression through ROCK, resulting in desensitization and internalization of the receptor to prevent over-excitation.

GPR39-1a constitutive activity likely promotes wound healing by decreasing oxidative, endoplasmic reticulum (ER), and mitochondrial stress and by decreasing inflammation [9,45]. Constitutive GPR39 activity transduced through the Gα12/13/Rho kinase pathway results in serum response element (SRE)-dependent transcription and cytoprotection in COS-7 and HEK-298 cells [9,46]. Similar observations were made in hippocampal cell lines overexpressing GPR39, in which resistance to oxidative stress from the endoplasmic reticulum (ER) and caspase pathways was mediated through the constitutively active, ligand-independent Gα13 signaling pathway and SRE-dependent gene expression (Figure 2; [41,42]). GPR39 constitutive activity has been detected in monkey COS-7, human HEK-293 cells, and hamster CHO cells (Table 1B, [9,11,25,26,27,47,48]).

## 7. Deorphaned Endogenous Ligands Implicate GPR39 in Moderating Neuro-Excitability and Vascular Tone through Gαq and/or Gαs Pathways

### 7.1. Obestatin Controversy

Obestatin (“obesity-related peptide”) became the first ligand proposed for GPR39 when it was isolated from rat stomach preparations and was reported to oppose ghrelin through GPR39, decreasing appetite [11]. However, this Obestatin binding to GPR39 proved non-reproducible by multiple groups [25,26,49] and was presumably due to contaminants in commercial obestatin preparations [27,50]. Nevertheless, a role for GPR39 in metabolism has been proposed by some groups based on increased weight and fat composition of some GPR39 knockout (KO) mouse lines (Table 2) [24,37].

### 7.2. Zinc Ligand-Activated Pathway Requires Specific Amino Acid Sites and Interacts with the Constitutive Pathway

Contemporaneous studies of GPR39 indicated that zinc was capable of activating the receptor [27]. Zinc is an essential dietary element that is highly regulated by the body, serving as a cofactor for many enzymes and transcription factors. Physiologically, zinc is important for immunoregulation, wound healing, epithelial barrier integrity, and nervous system functions. In cellular models, it promotes cell survival and proliferation through PI3, AKT, and MAPK pathways [51]. In the nervous system, zinc is known to alter the balance between neural excitatory and inhibitory signals by allosterically binding NMDA, GABA, and glycine receptors [52,53], but at the time of its discovery as an activator of GPR39, a potential role for zinc as a neuromodulator was not yet known.

ZnR triggers intracellular calcium signaling through GPR39 by binding to two His residues (His17 and His19) in the N terminus while simultaneously engaging Asp313 in the tridentate metal-ion binding site [27,43,54,55]. Mutation of His17 and His19 to Ala17 and Ala19 decreases zinc activation of GPR39 [43,56]. Such mutagenesis studies also illustrate the interdependence of ligand-dependent activation with GPR39 constitutive activity: when Asp313 is mutated to Ala313 at this tridentate metal-ion binding site of GPR39, zinc-dependent activation is eliminated, as expected, but surprisingly, also induces higher constitutive activity [43]. This functional site is highly specific since mutating adjacent His312 to Ala312 has no effect on ligand-activated or constitutive activity [43]. Specificity is further confirmed by two observations: GPR39 activation by zinc is not linked to stabilization of the receptor active conformation [43,56], and GPR39 receptor affinity to zinc can be modulated by extracellular calcium [57].

### 7.3. Zinc Induces Gαq and Gαs Cascade with Cross Modulation of the Constitutive Pathway

The following physiological observations contributed to the emergence of zinc as a candidate endogenous ligand for GPR39-1a: (1) Activation of GPR39 by zinc at physiologically relevant concentrations; for instance, low neural activation in mossy fiber synaptic terminals releases sufficient zinc concentrations to trigger GPR39 activation [22,54], (2) Zinc-associated GPR39 activation only in physiologic conditions (for example, zinc fails to activate GPR39 when pH falls below the life-threatening level of 6.5) [58], and (3) GPR39 isolated from different tissues responds to the physiologic level of zinc in that local tissue environment [23,26,27]. On the other hand, GPR39-1b showed no response to zinc in a cell-culture in-vitro calcium mobilization assay, suggesting it is not a functional zinc receptor [21].

Several intracellular pathways have been linked with GPR39 activation and are summarized in Figure 2. Studies utilizing CNS tissue or cell lines have shown that GPR39 knockdown or loss decreases zinc-triggered responses in neuronal cell lines [22,38,54]. Furthermore, pharmacological inhibitors of Gαq, inositol 1,3,4-triphosphate (IP3), and phospholipase C (PLC) pathways in hippocampal slices in the presence and absence of a zinc chelator (calcium disodium ethylenediamine tetraacetate) suggest that zinc-activated Gαq signaling is responsible for activating PLCβ to induce thapsigargin-dependent intracellular calcium release from ER stores [54]. Furthermore, this is mediated by the IP3 pathway, inducing extracellular-signal-regulated kinase (ERK) phosphorylation, calcium-calmodulin-dependent protein kinase activation, and diacylglcerol (DAG)-mediated phosphokinase C (PKC) and mitogen-activated protein kinase (MAPK) increase [8,23]. GPR39 stimulation by zinc also activates the Gαs pathway, which leads to cyclic adenosine monophosphate (cAMP) increase and phosphokinase A (PKA) activation. Both of these pathways lead to increased cAMP response element-binding protein (CREB) phosphorylation and cAMP response element (CRE)-dependent transcription and results in increased brain-derived neurotrophic factor (BDNF)/ Tropomyosin receptor kinase B (TrkB) in neurons. The latter pathway has been implicated in a variety of neuronal functions, including synaptic plasticity and remodeling, neuronal growth, circadian entrainment, improved memory, and protection against neuronal cell death and depression [26,45,59,60,61]. Outside the nervous system, the ligand-activated Gαq pathway is also linked to downstream extracellular signal-regulated kinase (ERK) 1/2 activation and phosphorylation of protein kinase B/Ak-strain transforming pathways (PKB/AKT) via MAP and phosphoinositide 3 (PI3) kinase to promote cell proliferation and survival in colon epithelial cells via CRE-dependent transcription [23,62,63,64]. 

While the Gαs and Gαq pathway are ligand-dependent, Gα12/13 is constitutively active through RhoA and serum response element (SRE) transcription (Figure 2; [15]). Cross-modulation between the constitutive and ligand-activated pathways can occur downstream of Gα activation [21]. Protein kinase A inhibitor beta (PKIB) was identified in a yeast 2-hybrid screen for interactors of the cytosolic GPR39 C terminus and appears to act in the Gα12/13 pathway to promote RhoA mediated SRE/serum response factor (SRF)/pigment epithelium-derived factor (PEDF) transcription through the constitutive pathway in the absence of zinc. However, upon zinc binding to GPR39, PKIB dissociates from GPR39 and no longer acts to promote the constitutive Gα12/13 pathway, but instead inhibits PKA in the Gαs pathway as a potential negative feedback loop to prevent zinc-mediated CRE over-excitation by ligand-bound GPR39 [15,46].

### 7.4. Eicosanoid Binding Implicates GPR39 Activity in Regulating Vascular Tone

Screening for the receptor of 14,15-epoxyeicosatrienoic acid (EET), a vasodilatory eicosanoid, revealed that GPR39 may serve as a dual-ligand eicosanoid receptor in microvascular smooth muscle cells (mVSMCs), sensing both the vasodilating 14,15-EET and the vasoconstrictive eicosanoid 15-hydroxyeicosatetraenoic acid (HETE). A chemical genetic approach initially identified GPR39 as a candidate 14,15-EET receptor [5], a result supported by RNAi knockdown in mVSMCs, computational modeling, and biochemical binding and signaling assays. Functionally, GPR39 stimulation by 15-HETE increases cytosolic calcium in VSMC cultures, a response that is eliminated by pre-application of 14,15-EET, suggesting opposing regulation by the two eicosanoids. Ex vivo Langendorff heart perfusion experiments further substantiated a physiologic link between GPR39 signaling and these eicosanoids. Specifically, coronary perfusion pressure increases in response to 15-HETE exposure, suggesting vasoconstriction, an effect that is eliminated in GPR39-null hearts or when 14,15-EET and 15-HETE are applied simultaneously. This observation suggests that GPR39 acts as a dual-ligand receptor of opposing eicosanoids to mediate the balance between vasoconstriction and vasodilation in mVSMCs. Interestingly, the effects of both 14,15-EET and 15-HETE are potentiated by zinc concentrations too low to activate GPR39 alone, indicating that zinc acts as a positive allosteric modulator (PAM) for eicosanoid binding. This PAM role for zinc has also been reported for certain synthetic agonists [6].

### 7.5. Synthetic Ligands, Biased Agonism, and Receptor Desensitization

A number of GPR39 synthetic ligands have emerged in recent years that exhibit biased agonism and ligand-specific activation of Gαs, Gαq, and Gα12/13 pathways [65]. These agonists include TC-G 1008 (GPR39-C3 or C3) [66] and GSB-118 [67], as well as LY2784544 and GSK2636771 [6]. These synthetic GPR39 agonists have been investigated for their therapeutic potential. GPR39-C3 is orally bioavailable and has been evaluated as a treatment for type 2 diabetes through its ability to increase GLP-1 (glucagon-like peptide 1) [66]. Some synthetic agonists (LY2784544 and GSK2636771) have been evaluated in animal models, with ongoing human trials in myeloproliferative disorders and cancers ([65,68]; www.clinicaltrials.gov available on 30 July 2021; GPR39-C3 has also been investigated for its antidepressant potential in animals [61].

Whether or not these therapeutic trials are ultimately successful, synthetic GPR39 ligands have been useful in elucidating the multiple activation cascade and negative feedback pathways for GPR39. In HEK293 cells, GPR39-C3 was shown to activate cAMP via Gαs and IP1 accumulation via Gαq and SRF/SRE dependent transcription downstream of Gα12/13 downstream, replicating the effects of both zinc-dependent and constitutive activity on GPR39 [67]. Furthermore, while GRP39-C3 can induce β-arrestin activity, β-arrestin does not appear to be responsible for GPR39 desensitization following C3 activation. Instead, GPR39-C3 exhibits a Rho-Kinase-dependent negative feedback on receptor activity upon repeated application, resulting in GPR39 internalization and desensitization [67]. GSB-118 activates cAMP through Gαs and β-arrestin without any Gα12/13-dependent SRF/SRE activity or desensitization upon repeated application, demonstrating ligand-selective signaling [67]. GPR39-activated pathways receive negative feedback from Rho Kinase via the Gαq cascade, resulting in ERK1/2 inactivation [67,69]. Such feedback mechanisms may protect cells from excitotoxicity through GPR39. For the remainder of this review, we will focus on GPR39’s roles in the CNS; for a comprehensive review of GPR39 pharmacology, please refer to Laitakari and colleagues’ excellent article [68] in a prior issue of this journal.

## 8. Role of GPR39 in Nervous System Homeostasis

### 8.1. GPR39 Tempers Neuroexcitability in Neurotransmission

Distinguishing the contribution of GPR39 in the CNS is complicated by a myriad of reports focused on zinc in normo- and patho-physiology. The difficulty in assigning particular observations to GPR39 activity arises from the variety of roles played by zinc in cellular physiology, though this has been improved through genetic loss-of-function experiments involving GPR39. The data on the role of zinc in neuroprotection is inconsistent, perhaps reflecting region-specific effects. Zinc is crucial for brain development, neurogenesis, and neurotransmission [52]. While ambient synaptic zinc is too low to regulate NMDA channels, activity increases zinc released from presynaptic glutamatergic vesicles and results in zinc levels sufficient to inhibit NMDA channels and modulate GABA and glycine receptor activity, thus, modulating synaptic integration and neuroplasticity [53]. This modulatory activity likely plays an essential role in setting the excitatory tone of neuronal circuits [53]. Zinc-dependent excitatory moderation requiring GPR39 has been demonstrated in hippocampal circuits. In mouse brain slices, mossy fiber stimulation triggers SNARE-dependent zinc release during synaptic transmission, activating postsynaptic GPR39-mediated increase of intracellular calcium increase in post-synaptic CA3 hippocampal cells [70]. This intracellular calcium release upregulates K-dependent Cl^−^ efflux in the postsynaptic cell through increased expression of KCC2 (K+/Cl−) cotransporter in the postsynaptic membrane to increase inhibitory tone and prevent excitotoxicity [54,70,71]. In rat and mouse cortical neuron cultures, this zinc- and calcium-dependent increase in KCC2 activity requires presynaptic zinc release and post-synaptic GPR39 expression; indeed, KCC2 upregulation is not observed when hippocampal slices from GPR39 KO were used [22]. Interestingly, this effect does not appear to involve post-synaptic Gαq cascade, suggesting alternative Gα pathways for intracellular calcium increases mediated by GPR39 [71].

Conversely, experimentally decreasing KCC2 in cultured hippocampal neurons increases intracellular Cl−, leading to impaired GABAergic inhibition [72] that is GPR39 dependent. Furthermore, post-synaptic calcium release was suppressed in the presence of a zinc chelator or when the synaptic zinc transporter ZnT3 was knocked out [22,54,73]. Thus, GPR39 has a role in inhibitory neurotransmission that is dependent on extracellular synaptic zinc levels that are, in turn, increased by synaptic activity [74]. Similar observations for GPR39’s role in activity-dependent inhibition have been made in the auditory brainstem and dorsal cochlear nuclei where GPR39 is expressed [75].

Another mechanism of GPR39-mediated modulation of neuroexcitatory tone was observed in hippocampal and auditory synapses where GPR39 triggers synthesis of endocannabinoids 2-arachidonoyl glycerol (2-AG) in postsynaptic cells, decreasing the probability of presynaptic glutamate release through cannabinoid receptor type 1 (CB1R) activation in the presynaptic membrane and, thus, decreasing synaptic strength [75,76,77]. Such zinc-mediated 2-AG activity was absent in GPR39 KO mice [75].

In summary, current data suggest that zinc acts on GPR39 to upregulate KCC2 and 2-AG expression to maintain homeostatic adaptation to potentially excitotoxic stimuli by increasing inhibitory tone. These findings demonstrate a critical role for GPR39 in neurotransmission and in the control of baseline excitatory tone. Thus, GPR39 may potentially contribute to disorders of neural circuit over-excitability, such as epilepsy and chronic pain. Pharmacologic targeting of GPR39 may prove effective in disorders characterized by dysregulated neural circuits and neurotoxicity from hyperexcitability, with potential for modulating epilepsy, inflammation, pH dysregulation, chronic pain, and neuropsychiatric disorders.

### 8.2. GPR39 in pH Homeostasis

Zinc activation of GPR39 contributes to pH homeostasis by upregulating the Na+/H+ exchanger (NHE1) in response to increased cytoplasmic pH [23,63,78]. While first observed in colonocytes and keratinocytes, this homeostatic mechanism is particularly pertinent to neurons as repeated activation results in intracellular acidification, changing neuronal excitability [23,79]. GPR39’s reported pH-sensitivity may contribute to regulating this process. Initially, increased postsynaptic GPR39 activity following repeated neural stimulation should result in increased NHE activity, decreasing intracellular metabolic acidification [79]. However, during ischemic glutamate release, NHE upregulation would continue, contributing to increased extracellular acidification of the neuronal surface, thus, increasing tissue acidosis and neural injury. Interestingly, this increasing extracellular acidity would ultimately reduce GPR39 activity since the receptor has been observed to be nonfunctional at pH < 6.5, attenuating NHE upregulation to protect ischemic neurons [23,80]. Such a negative feedback mechanism would allow GPR39 to maintain cellular physiologic pH and serve as a homeostatic neuroprotectant.

### 8.3. GPR39 Regulates Inflammation and Vascular Pathology

GPR39 contributes to inflammatory cascade regulation. In systemic infections, septic shock is associated with very high mortality due to multi-organ system failure caused by microvascular collapse via capillary leakage. In a mouse model of lipopolysaccharide (LPS)-induced inflammatory sepsis, the synthetic agonist GPR39-C3 increased production of the anti-inflammatory cytokine interleukin (IL)-10, resulting in improved survival that was dependent on GPR39 expression [81].

Recently, GPR39 was found to be expressed in human microglia, the central nervous system’s main immune surveillance cell [7], and the density of GPR39-expressing microglia is higher in post-mortem brains from individuals with a history of vascular cognitive impairment (VCI). In the same study, pericyte-like peri-capillary cells also demonstrated GPR39 immuno-reactivity, although the density of these peri-capillary cells did not differ between brains of individuals with and without VCI. The expression of GPR39 in these perivascular cells suggests GPR39 may play a role in microvascular blood flow in the brain and may contribute to VCI as neurons die from poor vascular support with increasing age and risk factors.

Outside of the central nervous system, GPR39 has been investigated in the vascular system for its regulation of inflammatory and proliferative tone in the setting of hypertension, turbulent blood flow, and atherosclerosis. Atherosclerosis begins via cell adhesion molecules triggered monocyte adhesion to vascular endothelial cells, causing further inflammation associated with calcification and vascular remodeling [82,83,84]. Areas of turbulent flow and increased mechanical stress display vascular smooth muscle cells that spontaneously dedifferentiate, downregulating contractile proteins and upregulating pro-proliferative, pro-migratory, and pro-inflammatory markers with enhanced matrix synthesis [82]. In a murine model of atherosclerosis, GPR39 mRNA expression was found to be increased in dedifferentiating VSMCs [82]. This expression is influenced by the disease process since dedifferentiated vSMCs from non-atherosclerotic mice exhibited greater GPR39 upregulation. Interestingly, GPR39-C3 treatment attenuates oxidized low-density lipoprotein (ox-LDL)-induced attachment of monocytes to endothelial cells by inhibiting p65/nuclear factor kappa-light-chain-enhancer of activated B cells (NF-κB) transcription, attenuating the first step of inflammatory plaque formation and atherosclerosis [84]. In primary human aortic VSMCs and mouse vascular calcification models, zinc supplementation upregulates tumor necrosis factor alpha induced protein 3 via GPR39, which suppresses NF-κB and blunts phosphate-induced vascular calcification [83]. Endothelial cells in human coronary blood vessels and mouse primary endothelial cells have also been shown to exhibit GPR39-dependent, zinc-triggered, calcium response via Gαq-PLC pathway to increase vascular cell growth and survival. This is mediated through the cAMP/Akt signaling cascade, resulting in increased platelet derived growth factor subunit A (PDGFA) receptor and vascular endothelial growth factor (EGF) A expression, which is critical for angiogenesis and vascular remodeling [85].

Vascular diseases, such as those described above, contribute to ischemic stroke and vascular cognitive impairment through the imbalance of nutrient supply to neural metabolic demand, resulting in neuronal cell death. Interestingly, extracellular zinc is neurotoxic, and the massive release of synaptic zinc during brain ischemia induces neuronal cell death [85,86]. It is likely that GPR39 plays a role in modulating neuronal cell death from such zinc toxicity. Whether it is the result of a large vessel stroke or multiple small vessel strokes, GPR39 may act to bind zinc and decrease neurotoxicity in this context.

## 9. Targeting GPR39 as a Therapy for Neurological and Neuropsychiatric Disorders

GPR39 is expressed in regions implicated in Alzheimer’s disease, epilepsy, and neuropsychiatric disorders, such as the frontal cortex, amygdala, and hippocampus [45]. Recent studies of GPR39 in the CNS support a disruption in the receptor’s homoeostatic role as a factor in pathogenesis.

### 9.1. Epilepsy Treatment

The importance of GPR39 in neurotransmission and the maintenance of hippocampal baseline excitatory tone positions it to play a role in epilepsy. Hippocampal slices from GPR39 KO mice do not show increased zinc release or KCC2 changes in response to stimulation, and this is associated with increased seizure-like gamma oscillatory activity [45,70,76]. Loss of synaptic zinc is also associated with increased seizure activity, especially febrile seizures, which may be mediated through GPR39 [23,87,88,89]. In vivo, rats fed a zinc-deficient diet for 4 weeks showed increased cognitive deficits and decreased GPR39 brain expression after induction of developmental seizures compared to rats fed normal zinc diets. These deficits can subsequently be partially rescued after eating zinc supplemented diets for 4 weeks with normalization of GPR39, ZnT3 (zinc transporter), and myelin basic protein expression (representing oligodendrocyte contributions to neuronal membrane integrity and axonal regeneration) in the hippocampus [90]. Indeed, GPR39 KO animals have increased susceptibility to stage 5 seizures that manifests as loss of posture and status epilepticus following kainate-induced seizure activity [76]. While increasing zinc levels may improve seizure outcomes via GPR39 [90], the synthetic agonist GPR39-C3 does not appear to be a chemo-preventative as it does not increase the current threshold required for seizure induction using the maximal electroshock seizure threshold test in rats [76].

### 9.2. Chronic Pain

Eicosanoids and zinc are both implicated in the pain signaling cascades and may be mediated through GPR39. In a study of orofacial and headache disorders after sensitization of trigeminal primary afferents with Complete Freund’s Adjuvant injection, rats exhibited orofacial inflammation, associated with upregulation of GPR39 mRNA in ipsilateral vs. contralateral trigeminal ganglia, during the 3–7 day period when mechanical allodynia was greatest [91]. In this case, GPR39 upregulation may be serving a neuroprotective, anti-inflammatory role in response to inflammation, partially mitigating the pain hypersensitivity.

### 9.3. Alzheimer’s Disease and Vascular Dementia

Numerous lines of evidence indirectly implicate GPR39 in Alzheimer’s disease (AD). However, it is important to mention that zinc perturbation may have GPR39-independent effects on AD development and progression. Specifically, AD and amyloid-β plaques have been shown to sequester and lower zinc levels [92], which may affect GPR39 function. The high level of hippocampal GPR39 expression, and GPR39’s participation in hippocampal zinc neurotransmission, make both a candidate in Alzheimer’s disease (AD) pathogenesis and potential therapeutic targets [12,54]. The molecular signaling downstream of GPR39 activation converges with pathways that have been implicated in AD. Specifically, activated GPR39 upregulates KCC2, and amyloid-β-induced zinc dyshomeostasis results in blocked postsynaptic GPR39 and, consequently, down-regulated KCC2, increasing neuroexcitatoxicity and neural death [15]. ERK1/2 activation from this signaling pathway has been shown to inhibit clusterin, a protein associated with Alzheimer’s disease [92]. Indeed, the AD11 mouse model of Alzheimer’s disease demonstrated that neurons high in Aβ plaques had downregulated KCC2 mRNA, potentially from decreased GPR39 activation, and exhibited such predicted depolarizing GABA_A_ currents in CA1 pyramidal neurons [15]. In mouse models, increasing intracellular zinc (and by extension zinc-induced GPR39 activity) using clioquinol or PBT2 has been demonstrated to decrease Aβ levels, restore postsynaptic GPR39 protein expression, and improve cognitive outcomes [52]. While Aβ burden is directly correlated with dementia severity and anti-correlated with zinc levels and clearance in AD patients [93,94], future studies will be required in human AD brains and mouse models to assess whether this is due to decreased GPR39 activity and whether restoring or increasing GPR39 activity can restore homeostasis and slow AD progression. In vitro, GPR39-induced calcium responses in primary neuron cultures decreased in the presence of Aβ and resulted in decreased MAPK activation that has been implicated in Alzheimer’s pathology [92]. In animal models, while supplementation with Zn and Se decreased mitochondrial protein collapse, ROS production, and lipid peroxidation while increasing brain mitochondrial glutathione peroxidase and catalase with improved cognitive performance, it did not significantly change GPR39 expression [95]. Indeed, clinical trials using zinc for AD in humans have been inconclusive [96], which could be due to uncorrected GPR39 dysfunction despite supplemental zinc.

Vascular cognitive impairment (VCI) is the second leading cause of dementia after AD. VCI is difficult to distinguish from AD since both are progressive dementias, and AD requires a post-mortem exam for proper diagnosis. However, while AD postmortem brains demonstrate the classic tau tangles and Aβ plaques, VCI brains tend to show white matter lesions on MRI and microvascular pathology [83,84]. An intriguing question is whether vascular cognitive impairment acts through GPR39 while Aβ-associated AD is more independent of GPR39. Postmortem human brain samples from cognitively intact individuals and patients with mild cognitive impairment (MCI) were recently evaluated using GPR39 immunohistochemistry. GPR39 was found to be expressed with equal density between diseased and control brains and appeared to colocalize with cells resembling activated microglia and pericytes more densely in brains with vascular dementia, though the overall expression density was the same in both populations [7]. Indeed, diseased brains from MCI patients had increased GPR39-expressing microglia density compared to controls. Of note, no neuronal staining of GPR39 was seen in these human brains. Though homozygous GPR39 SNP carriers were only present in the MCI group, and while the MCI group had higher white matter hyperintensity burden, the N (=5 homozygous GPR39 SNP carriers out of 78 total samples) was low and unable to statistically demonstrate correlation in this study. Thus, GPR39 SNPs may be a future biomarker in aging-related VCI and may play a role in neuroinflammation through microglia expression [97], as well as a role in brain perfusion dysfunction through pericyte expression [98,99,100]. The potential of GPR39 SNP correlation with white matter hyperintensity burden also suggests that a premortem MRI can help diagnose early, interventionable, pre-symptomatic pathological changes associated with VCI [7]. Thus, the role of GPR39 in VCI should warrant more investigation, particularly whether GPR39 expression is a protective reaction to inflammation. As microglial GPR39 expression and signaling transduction pathways become better understood, the receptor becomes targetable as a potential mechanism to decrease dementia-related neuroinflammation.

### 9.4. Neuropsychiatric Disease: Anxiety, Depression, Addiction

Many pharmacologic neuropsychiatric therapies have the goal of “rebalancing” monoaminergic or glutamatergic signaling. GPR39’s ligand, zinc, has been repeatedly studied as a potential treatment for depression and anxiety. Indeed, zinc deficiency correlates with increased neurologic and psychiatric disorders [101]; since GPR39 expression decreases with zinc deficiency, GPR39 is likely implicated in the pathogenesis of these disorders. Thus, it is not surprising that the putative role of GPR39 in neuropsychiatric disorders have been investigated in animal models.

#### 9.4.1. Anxiety

GPR39 is highly expressed in the hippocampus and amygdala, regions that encode fear, anxiety-provoked behavior, and stress memory [10,38,102,103,104]. GPR39 KO mice demonstrate increased anxiety behavior relative to control littermates [38]. Using the synthetic agonist GPR39-C3, increased GPR39 activity was demonstrated to have antidepressant-like properties in mice undergoing forced swim and tail suspension challenges, and GPR39-C3 also exhibited anti-anxiolytic (potentially sedative) effects in the elevated plus maze and light-dark tests. These behavioral changes were substantiated histologically by increased GPR39 expression in the hippocampus and upregulated BDNF expression after agonist administration [105]. More directly, Ishitobi and colleagues [106] infused antisense oligonucleotides of the GPR39-1b splice variant into rat cerebral lateral ventricles and produced anxiolysis in an elevated plus maze and black and white box test.

#### 9.4.2. Depression

GPR39 expression levels are decreased in the hippocampus and cortex of postmortem suicide victims, paralleling decreased GPR39 expression levels observed in rodents fed zinc deficient diets that produced depressive-like behavior [107]. In contrast, rats with depressive behaviors due to olfactory bulbectomy showed increased GPR39 in hippocampus, perhaps suggesting a regional difference in GPR39-related depression or a compensatory or protective response in hippocampus to the depressive state [107]. GPR39 expression by Western blot in the frontal cortex of mice was upregulated after a 14-day treatment with selective monoaminergic inhibitor antidepressants, but not with a broader acting tricyclic antidepressant [38,103,107,108]. GPR39 deficiency may abolish these monoaminergic-based antidepressant effects since GPR39 KO mice do respond to treatment with the selective serotonin reuptake inhibitor (SSRI), escitalopram, in a forced-swim depression assay [61,102]. These findings indicate that GPR39-dependent anti-depressive effects may be pathway or cell-type specific, with three main modes of action currently under investigation: modulation of zinc levels, neurotransmitter signaling, and neuroinflammation. Evidence for each mechanism is described below.

##### Depression and Zinc Agonism of GPR39

BDNF has been widely implicated in depression disorders, and zinc activation of GPR39 leads to increased BDNF via the Gαs pathway and CRE-dependent transcription [101]. Chronic antidepressant treatment in zinc deficient mice also upregulates GPR39, which appears to act through the CREB/BDNF/TrkB pathway via Gαq for its antidepressant effects [9,101,103]. A 6-week zinc deficient diet produces increased immobility in a forced swim test with concurrent decreased GPR39 and BDNF protein expression in the frontal cortex [109]. A similar effect on GPR39 expression is also observed in the chronic restraint stress model of depression, where mRNA levels decreased for GPR39, BDNF, and CREB; interestingly, changes in behavior, GPR39 expression, and BDNF expression were rescued by 30 mg/kg of zinc and 20 mg/kg of the antidepressant imipramine [110].

Since GPR39 agonism exhibits signaling bias, not all GPR39 agonists have zinc’s anti-depression qualities. The synthetic GPR39 agonist GPR39-C3 was shown to decrease immobility at the time of acute injection in a forced swim test but was ineffective at improving tail suspension or locomotor impairment, even though it increased GPR39, CREB, BDNF, and TrkB levels in the hippocampus after chronic administration [103,105]. Chronic treatment with GPR39-C3 also appeared to decrease immobility time with a trend for increased BDNF expression but with no corresponding increase in brain or serum zinc levels [61]. Interestingly, in one study, zinc appears to have no effect on depression behavior alone but appears to potentiate the effects of the SSRI fluoxetine in the GPR39/BDNF-mediated treatment of depression from chronic mild stress [111]. Thus, GPR39 may mediate depression development and progression both dependent and independent of its role as a zinc receptor and may begin to explain why targeting zinc levels alone, without concurrently targeting GPR39, has not consistently been successful in ameliorating depression or depression symptoms.

##### Neurotransmission Modulation (Monoaminergic and Glutamatergic Systems)

Two-week treatment with escitalopram or the SSRI reboxetine increased GPR39 protein expression in the mouse frontal cortex with improved behavioral parameters [40,107,108]. Critically, no improvement in forced swim test scores was observed in GPR39 KO mice in response to either treatment, indicating that GPR39 signaling is required for these effects. Indeed, there is a growing link between GPR39 and the 5-HT system. When a tyrosine hydroxylase inhibitor (alphaMT, preventing catecholamine synthesis (NE/DA) pathway) and a tryptophan hydroxylase inhibitor (pCPA, preventing serotonin synthesis (5-HT) pathway) was given to mice, GPR39 expression was reduced in the hippocampus, and GPR39 KO mice were observed to have decreased monoamine precursors, tyrosine and tryptophan, in their hippocampus [102]. Interestingly, the alphaMT treatment also seemed to upregulate GPR39 in the mouse frontal cortex after 3 treatment days, despite its opposite effect on hippocampal GPR39 expression after 10 days [102], again highlighting regional specificity of GPR39 responses. At the receptor level, the serotonergic 5-HT1a was observed to heterodimerize with GPR39 and with GPR39-GalR1 (GPR39-galanin receptor dimer) complex [112]. This suggests that GPR39 may be regulated by 5-HT receptor signaling through dimerization to increase constitutive activity via the SRE. In zinc deficient animals, the heterodimer 5-HT1a-GalR1 is pathologically observed due to a decrease in GPR39 expression and, thus, may reflect a phenotype of the zinc deficiency [113,114].

Perturbation of glutamate signaling in depression models also reveals additional aspects of GPR39’s effect. NMDA glutamate receptor antagonists decrease immobility time in a forced swim test in GPR39 KO mice, while both monoamine-based antidepressants and NMDA antagonists worked in WT animals [38]. This suggests GPR39 is necessary for monoaminergic antidepressant activity but not necessarily required for the antidepressant activity exhibited by NMDA glutamate receptor antagonists. GPR39 stimulation also increases KCC2, the neuron-specific transporter that maintains low intracellular chloride concentration and stimulates GABAergic transmission, maintaining the balance between excitatory and inhibitory states [22,38,102]. Lowered GPR39 activity could, thus, contribute to the increased glutamate concentrations observed in the serum and brain tissue of depressed patients. Decreased GPR39 activity may also increase glutamate concentrations from reduced postsynaptic 2AG synthesis and, subsequently, increased CB1R-mediated glutamate release, ultimately resulting in neuroexcitotoxicity [61,75].

##### Depression Interplay with Neuroprotection and Inflammatory Pathways

Dysregulated immune system function and neuroinflammation have recently been implicated as a factor in neuropsychiatric disorders. Along with increased depressive behaviors, one line of GPR39 KO mice also had reduced thymus weight, reduced splenocyte viability, decreased proliferative immune responses, and decreased interleukin (IL)-1b/IL-6 release after LPS-induced sepsis; this parallels the components of immune dysregulation observed in depression patients [39]. Aspects of the peripheral nervous system (PNS) responsible for controlling gastrointestinal function have also been implicated in precipitating neuroinflammation. One study, for instance, linked increased IgA and IgM against gut-commensal, Gram-negative bacteria to a subset of depression patients with poor epithelial barrier formation at tight functions [115]. Since zinc acts through GPR39 to enhance colonic cell survival and tight junctions, GPR39 may regulate this inflammatory response through Gα13/RhoA/SRE-dependent transcription to increase pigment-epithelium derived factor (PEDF) production, along with other cytoprotective factors [41,116,117]. Increasing GPR39 expression in a hippocampal-derived cell line conveyed increased protection against oxidative stress, ER stress, and BAX-mediated cell death [41], suggesting GPR39 may protect against the neurotoxic stresses that may underlie neuropsychiatric disorders [61]. A cortisol-induced cell injury model has been used to evaluate the neuroprotective effects of GPR39, in which hippocampal neurons were assessed for mitochondrial function and apoptosis activation. The study tested both decreased GPR39 expression (siRNA knockdown) and increased GPR39 signaling (via GPR39-C3 treatment). Indeed, GPR39 was found to be neuroprotective via the CREB-BDNF expression axis by inhibiting pro-apoptotic proteins, such as caspases, and by upregulating anti-apoptotic proteins, such as BCL-2 [118].

#### 9.4.3. Addiction

GPR39 also likely participates in addiction modulation due its notable expression in the nucleus accumbens [119]. In a study on rhesus macaques, the DNA methylome of the nucleus accumbens core after chronic alcohol use showed GPR39 hypermethylation, resulting in downregulated GPR39 expression in heavy-drinking macaques compared to normal drinkers [119]. In rodent models, the GPR39 synthetic agonist GPR39-C3 was capable of decreasing ethanol intake in mice when acutely dosed, without affecting their total fluid intake, locomotor activity level, or saccharin preference in a two-bottle choice model. In fact, repeated dosing of GPR39-C3 was able to prevent ethanol use escalation, an effect that was reversed with agonist washout [120]. Mechanistically, GPR39 activation by GPR39-C3 was associated with GPR39 and BDNF expression changes and changes in glutamate release in the nucleus accumbens [119]. This confirmed the data in an earlier study in which rhesus macaques that self-administered 4% alcohol over 12 months demonstrated 17 differential CpG-rich methylation regions, including hypermethylated GPR39 exon 1, and which showed, by RT-PCR, decreased expression levels that were anti-correlated to methylation level in the nucleus accumbens [120]. Since GPR39 expression is closely tied to zinc levels, with zinc deficiency commonly observed in alcoholics, the downregulation of GPR39 may reflect decreased zinc levels. This may ultimately result in decreased GABA_A_R expression and increased glutamate release via GPR39’s actions on KCC2 and 2-AG synthesis, respectively, thus, increasing the excitatory tone of the circuit [120]. This may have profound implications for ethanol dependence and the increased potential for withdrawal seizures during attempts to cease ethanol consumption.

### 9.5. Neuroendocrine Influences on the Nervous System

GPR39 has been investigated for its contribution to the endocrine system [11]. However, GPR39 KO mice have not been reported to have a diabetic phenotype and appear to have normal insulin secretion under baseline conditions [25,36]. Other studies demonstrated that older GPR39 KO mice fed a high sucrose or high fat diet may have higher glucose and decreased insulin secretion compared to their wild type littermates [35,36,121]. In mice with streptozocin-induced diabetes, overexpression of GPR39, specifically in beta islet cells, protected against gradual hyperglycemia, even though GPR39 overexpression without streptozocin induced diabetes and appeared to impair glucose tolerance [122]. Again, this suggests a homeostatic role of GPR39 in glucose tolerance and insulin secretion and indicates that additional studies will be needed to resolve the roles played by GPR39 along the neuro-endocrine axis.

## 10. Looking toward Future Research into GPR39’s Role in Neurovascular Pathology

**Figure 3:**
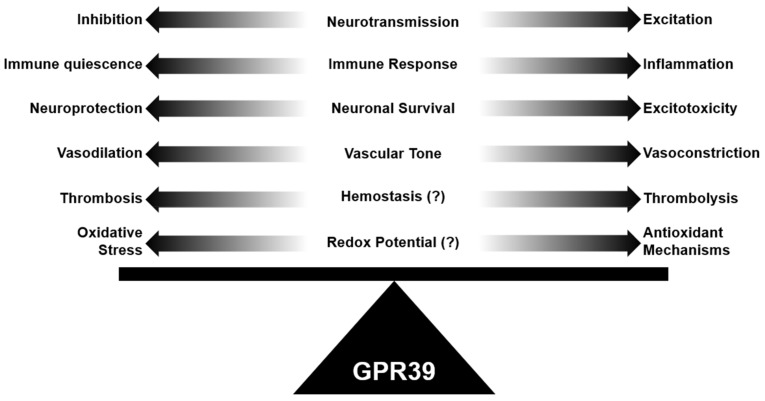
GPR39 is implicated in neurovascular and neuropsychiatric homeostasis. GPR39 plays a role in balancing neurotransmission, immune response, neuronal cell survival, vascular tone, and redox potential, suggesting it is uniquely positioned to promote homeostasis in the neurovascular and neuropsychiatric system. Disruption of GPR39 function may lead to dyshomeostasis and neuropathology.

In this review, we have discussed the unique role of GPR39 in maintaining physiological brain homeostasis in many components of the neurovascular system. We have summarized how GPR39 may contribute to neuropathology through diverse agonist binding and the likely role of zinc as a positive allosteric agonist. Specifically, GPR39 appears to have a consistent role in maintaining brain homeostasis, whether it is through maintaining excitatory/inhibitory tone in the neural circuit, maintaining vascular tone in the microcirculation, or decreasing inflammatory tone (Figure 3). All three mechanisms have profound consequences in the brain and its neurovascular system since neurotoxicity can occur from over-excitation, inflammation, or poor vascular supply. Future investigation of GPR39 will likely include GPR39 cell-specific deletion studies in components of the neurovascular system, expanded biochemical assessment of the molecular basis of receptor activation via both natural and synthetic agonists of GPR39, and clearer understating of how GPR39 splice variants and dimerization partners shape GPR39-dependent physiology in the neural and vascular systems. A closer look at species-specific GPR39 expression patterns will also be important for translating animal studies to human therapies. Basic, translational, and clinical inquiries will be required to fully harness the therapeutic potential of GPR39 agonists in human neuropathology.

## Figures and Tables

**Table 1:** (**A**) Expression pattern of GPR39 in published studies. GPR39 shows variable expression in published literature dependent on splice variant, species, and detection method. (**B**) In-vitro GPR39 expression and signaling cascades.GPR39 over-expression (OE) in in-vitro systems generate G-protein signaling cascades.

(A)					
Organism	Detection Method	Probe Source	GPR39 Variant Detected	Expression Pattern	Publication Source
Human	Northern blot for RNA, probe using ^32^P- Labeled DNA fragment with complete open reading frame	Fetal human brain cDNA library, GPR39 isolated by rapid PCR in search for relatives of Growth Hormone Secretagogue Receptor and Neurotensin Receptor Type 1.	1a, 1b	Amygdala, caudate nucleus, corpus callosum, hippocampus, substantia nigra, thalamus, cerebellum, cerebral cortex, medulla, spinal cord, occipital pole, frontal lobe, temporal lobe	[8]
Mouse	In situ hybridization, mRNA	Mouse	1a, 1b. Probe does not overlap with LYPD1/ MGC29643 antisense gene	Amygdala, hippocampus (dentate gyrus, CA1, CA3), auditory cortex, layer 2 piriform cortex, ventral pallidum, inferior olive, NOT in hypothalamus	[10]
Rat	Quantitative RT-PCR (QPCR), in situ hybridization	Rat	1a	Very low expression in CNS; high expression in peripheral metabolic organs	[12]
Rat	Quantitative RT-PCR (QPCR)	Rat	1b	Widely expressed but low expression in cerebellum, cortex, pons, hippocampus, hypothalamus, striatum, amygdala, septum. 1b overlaps with antisense gene LYPD1, which is expressed strongly in all brain regions.	[12]
Mouse	Quantitative RT-PCR (QPCR),	Mouse	1a	Low levels in whole brain, septum, hypothalamus, hippocampus	[24]
Mouse	GPR39 promoter expressing LacZ instead of GPR39, no functional GPR39	Mouse	1a, 1b	Septum, hippocampus (dentate gyrus), amygdala (discrete cells), NO hypothalamic expression. Strong expression in small intestine nerve plexus.	[24]
Rat	Quantitative RT-PCR (QPCR)	Rat	1a, 1b	Pituitary, hypothalamus, cerebellum, cerebrum	[11]
Mouse	RT-PCR in GPR39 KO mice and WT littermates	Mouse	1a, 1b	Low brain expression	[25]
Rat	Quantitative RT-PCR (QPCR)	Rat	1a, 1b	NOT in pituitary or hypothalamus	[26]
Human	Protein— immunohistochemistry, antibody to GPR39 third extracellular domain	Anti-human antibody	1a	Staining in microglia and peri- capillary cells (pericytes), higher density of GPR39 expressing microglia in mild cognitive impairment brains	[7]
**(B)**					
**In vitro** **System**	**Intervention**	**GPR39** **Variant**	**Result**		**Publication** **Source**
CHO cells (hamster ovary), HEK293T cells (human kidney)	GPR39 cDNA expression	1a, 1b	Increased obestatin stimulation with cAMP production; GPR39 constitutively activates serum response element (SRE)		[11]
COS-7 cells (monkey kidney)	GPR39 expression from human stomach cDNA library	1a, 1b	Zn^2+^ stimulated Gs pathway and cAMP increase; high constitutive activity		[26]
HEK293T cells (human kidney)	GPR39 overexpression from human cDNA	1a, 1b	GPR39 constitutively active through SRE- mediated transcriptional activity		[25]
CHO-K1 cells (hamster ovary)	Human and mouse GPR39 cDNA (1a) from genomic DNA, rat GPR39 obtained by RT-PCR from cDNA of rat liver.	1a, 1b	Zn^2+^ identified as GPR39 agonist in fetal bovine serum peptide extraction, showed Zn mobilizes calcium through Gαq-PLC pathway for human, mouse, and rat GPR39.		[27]

**Table 2:** Summary of GPR39 knockout mouse models. Disruption strategy and observed phenotypes are detailed for each GPR39 knockout line with respective references.

Knockout Strain Name	Targeting Strategy	Phenotype Observed	Reference
Gpr39tm1Lex	Exon 1 replacement with selection cassette	Increeased body weight and fat composition, increased cholestrol levels, reduced hyperphagia after fasting.	[24]
		loss of hippocampal Zn-enhanced KCC2 activity and surface expression	[22]
		increased sensitivity to dextran sodium sulfate ulcerative colitis model and reduced rate of recovery	[33]
		Increase in Cholera-toxin induced intestinal fluid secretion	[32]
		Increased numbers of active osteoblasts, disorganized bone matrix deposition, down-regulation of collagen processing enzymes	[31]
Gpr39tm1Dgen	Exon 1 selection cassette knock-in	No effect on body weight or food intake	[25]
		Impaired glucose tolerance, decreased plasma insulin response	[35]
		Impaired insulin secretion	[36]
		Increased fat accumulation with high-fat diet, elimination of diet-induced thermogenesis	[37]
		Decreased TMEM16A current in small intestine fibroblast-like cells	[30]
		Resistance to monoamine-based antidepressants	[38]
		depressive-like behavior, reduced thymus weight; reduced splenocytes viability, reduced splenocytes proliferative response, increased IL-6 production	[39]
		Delayed wound healing	[34]
		lower hippocampal CREB and BDNF levels, depressive-like behavior and anxiety-like phenotype	[40]
Gpr39em1(IMPC)Bay	CRISPR Exon 1 Deletion	decreased bone mineral density	International Mouse Phenotyping Consortium (IMPC)

## Data Availability

Not applicable.

## Abbreviations

14,15-EET	14,15-epoxyeicosatrienoate, vasodilating eicosonoid
15-HETE	15-hydroxyeicosatetraenoate, vasoconstricting eicosonoid
2-AG	2-arachidonoyl glycerol, endocannabinoid
5-HT	serotonin
Aβ	amyloid beta
AML	acute myeloid leukemia
BDNF	brain-derived neurotrophic factor
cAMP	cyclic adenosine monophosphate
CAD	coronary artery disease
Ca levels	calcium levels
CBR1	endocannabinoid receptor 1
CHO	cell line derived from Chinese hamster ovaries
COS-7	cell line derived from kidney of African Green Monkey
CRE	cAMP response element (CRE) transcription factor
CREB	cAMP response element-binding protein (CREB)
DAG	diacylglcerol
ECL	extracellular domain
ERK	extracellular signal-regulated kinase
Gα12/13	G-protein inositol alpha 12/13, GPR39’s constitutively active signaling cascade
Gαq	G-protein inositol alpha q, zinc-activated signaling cascade through GPR39
Gαs	G-protein inositol alpha s, zinc-activated signaling cascade through GPR39
GnR	ghrelin receptor
GPCR	G-protein coupled receptor
GPR39	G protein coupled receptor 39, also known as ZnR (zinc receptor)
GPR39-C3	synthetic agonist of GPR39, also known as C3 or TC-G 1008
HEK293T	Cell line derived from human kidney
HT	hypertension
IL-10	interleukin 10
IP3	1,3,4-triphosphate
KCC2	K+/Cl-cotransporter
KO	knock out
LPS	lipopolysaccharide (LPS), bacteria-derived compound to induce inflammatory sepsis
Lung Cap	lung capacity
LYPD1	LY6/PLAUR domain containing 1 gene, antisense gene overlapping with GPR39’s second exon, overlaps with GPR39-1a but not GPR39-1b
MAPK	mitogen-activated protein kinase
MCI	mild cognitive impairment
mVSMCs	microvascular smooth muscle cells
NHE-1	Na+/H+ exchanger
NTSR1	Neurotensin receptor 1
OE	over expression
PAM	positive allosteric modulator
PI3	phosphoinositide 3
PKA	phosphokinase A
PKB/AKT	protein kinase B/Ak-strain transforming pathways
PKC	phosphokinase C
PKIB	Protein kinase A inhibitor beta
PLC	phospholipase C
sc-RNAseq	single cell RNA sequencing
SRE	serum response element, constitutive pathway of GPR39 signaling cascade
SNRI	serotonin and norepinephrine reuptake inhibitor
SSRI	selective serotonin reuptake inhibitor
TM	transmembrane domain
TrkB	Tropomyosin receptor kinase B
VCI	vascular cognitive dementia
VCID	vascular cognitive impairment and dementia
VSMC	vascular smooth muscle cell
WT	wild type
ZnT3	zinc transporter
